# The PCNA pseudogenes in the human genome

**DOI:** 10.1186/1756-0500-5-87

**Published:** 2012-02-06

**Authors:** Ivaylo Stoimenov, Anne Lagerqvist

**Affiliations:** 1 Department of Genetics, Microbiology, and Toxicology, Stockholm University, S-10691 Stockholm, Sweden; 2 Department of Genetics, Microbiology and Toxicology, Stockholm University, Svante Arrhenius väg 20C, S-10691 Stockholm, Sweden

## Abstract

**Background:**

The proliferating cell nuclear antigen (PCNA) is a key protein in the eukaryotic DNA replication and cell proliferation. Following the cloning and characterisation of the human PCNA gene, the question of the existence of pseudogenes in the human genome was raised.

**Findings:**

In this short communication we summarise the existing information about the PCNA pseudogenes and critically assess their status.

**Conclusions:**

We propose the existence of at least four valid PCNA pseudogenes, PCNAP1, PCNAP2, LOC392454 and LOC390102. We would like to recommend assignment of a name for LOC392454 as "proliferating cell nuclear antigen pseudogene 3" (alias PCNAP3) and a name for LOC390102 as "proliferating cell nuclear antigen pseudogene 4" (alias PCNAP4). We prompt for more critical evaluation of the existence of a PCNA pseudogene, designated as PCNAP.

## Findings

The proliferating cell nuclear antigen (PCNA) is a key protein in the eukaryotic DNA replication and cell proliferation. The first observation of PCNA as an antigen in sera, isolated from patients with systemic lupus erythematosus, started a new field of research [1]. Already in this pioneering investigation, the function of PCNA in cellular proliferation was revealed, however the true nature of the antigen was not known. The identification of PCNA as an auxiliary protein to DNA polymerase delta [2,3], placed PCNA as a protein of a vital importance and prompted the question of gene characterisation. In the same year, the human cDNA encoding the PCNA protein was cloned and characterised [4]. The molecular cloning of the human PCNA gene followed soon, hinting for a possibility for existence of a PCNA pseudogene in the genome [5]. The PCNA gene was mapped to a locus in human chromosome 20 [6,7]. An interesting serendipity of the gene localisation was the discovery of several related to PCNA loci, which were proposed to contain pseudogenes of PCNA [6,7]. Since then, several reports of PCNA pseudogenes have been published [5-9]. However, the current state and validity of those pseudogenes is an open question. There is considerable discrepancy between the major human-related databases and the experimental data. In this short communication we summarise the evidence for the PCNA pseudogenes and try to validate their status.

There are several milestone papers describing human PCNA pseudogenes, which for simplicity will be further referred to as roman numerals according to their order of publication [5-9].

The first paper (Paper I) characterises the PCNA gene, however, it mentions a possible PCNA pseudogene [5]. The follow-up paper from the same group (Paper II) describes this PCNA pseudogene in detail [6]. Paper I reports that in a total genomic EcoRI digest of human DNA, three distinct fragments can be detected by hybridization with a full length cDNA probe, derived from the human PCNA sequence. These fragments are with size 3.7 kb, 2.7 kb and 1.5 kb [5]. The larger fragments (3.7 kb and 2.7 kb) can originate from the PCNA gene, however, the smallest fragment (1.5 kb) was proposed to be a product of a PCNA pseudogene [5]. Indeed, in Paper II the existence of this the pseudogene was confirmed and its sequence was published [6]. The sequence lacks introns and possesses the characteristics of processed pseudogenes or nonviral retrotransposons [6]. This PCNA pseudogene was mapped to the human chromosome X in the region Xpter-Xq13. In Paper II, another possible locus for a PCNA pseudogene was hypothesised to exist, situated on the human chromosome 6. The authors show detection of a PCNA related sequence in one of the mouse-human somatic cell hybrid clones (N9) which retained human chromosomes 6, 7, 21 and partial 17 [6]. The presence of a PCNA related sequence on chromosome 6 was investigated in another of the authors' experiments, however, the vague and non-aligned hybridisation of a full length PCNA probe to chromosome 6, presented in the paper suggests merely an artifact [6]. An interesting conclusion from the results in the Paper II is that if there is a PCNA pseudogene on chromosome 6, it does not contain introns, as is the case with the PCNA pseudogene on chromosome X. The only strong evidence presented in the paper for existence of a PCNA pseudogene on chromosome 6 is the clone N9. However, in our opinion a careful characterization of the N9 clone is needed in order to exclude the presence of fragments of other human chromosomes (11, 20 and X). The argument for such characterization of the N9 clone is the next important paper (Paper III), which maps the chromosomal location of the PCNA gene and two of the PCNA pseudogenes [7].

Indeed, in Paper III, the presence of a PCNA pseudogene on chromosome 6 was not detected [7]. In Paper III the localisation experiments for the PCNA gene and its pseudogenes are conducted by using a probe, derived from a fragment of the human PCNA cDNA. The results revealed a presence of three loci, hybridising with the probe-the PCNA gene (chromosome 20), and two pseudogenes-on chromosome X and chromosome 11. The PCNA pseudogene on chromosome X is mapped in the region Xp11.3-Xp11.4, which not only confirms the location presented in Paper II, but gives a more precise location of the proposed PCNA pseudogene. The presence of a sequence which anneals with the probe on chromosome 11 in the region 11p15.1, represent another PCNA pseudogene.

The fourth publication (Paper IV) presents new PCNA pseudogenes [8], which are further characterised in the follow-up study by the same group (Paper V) [9]. Both papers IV and V supply strong evidence for the existence of PCNA pseudogenes on chromosome 4 in the region 4q24. The two sequences described in the paper have already been assigned names by HUGO, "proliferating cell nuclear antigen pseudogene 1" (PCNAP1) and "proliferating cell nuclear antigen pseudogene 2" (PCNAP2). The existence of PCNA pseudogenes on human chromosome 4 was proposed earlier by the same group in Paper IV, but the later paper presents a more detailed study and sequence of the PCNA pseudogenes. Webb et al. failed to detect PCNA pseudogenes on the human chromosome 4, possibly because of the limitation of the probe used [8]. In fact PCNAP2 is a fragment which contains sequences homologous to exon 4 and 5 of the PCNA gene [9].

In the HUGO database [10], maintained by HUGO Gene Nomenclature Committee (HGNC), there are currently three active entries for PCNA pseudogenes: PCNAP [11], PCNAP1 [12] and PCNAP2 [13]. The PCNAP, PCNAP1 and PCNAP2 are present as PCNA pseudogenes in the NCBI GENE database [14] together with LOC392454 and LOC390102 [15].

PCNAP1 and PCNAP2 refer to the PCNA pseudogene sequences published in Paper V.

**Figure 1:**
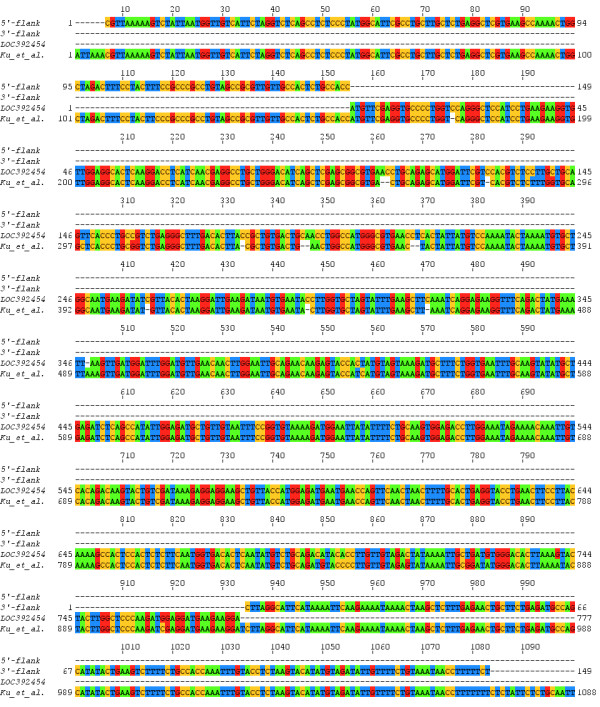
**Alignment of the sequences of the PCNA pseudogene published by Ku et al**. [**6**]**and LOC392454, including 5'- and 3'-flanking regions of LOC392454**.

The LOC392454 is placed in the same locus as the PCNA pseudogene, detected by Ku et al. (Paper II) and confirmed by Webb et al. (Paper III). We aligned the sequence, published by Ku et al. with the sequence of LOC392454 and found that they match very well (Figure 1). In the alignment we included two additional sequences, which flank LOC392454 in 5'- and 3'-direction, each spanning 150 bp. The match in the flanking sequences and the sequence of the PCNA pseudogene, described by Ku et al., was nearly perfect with only one mismatch (Figure 1). From the alignment, we could say that the sequence of LOC392454 is nearly identical with that published by Ku et al. (Figure 1). A simple pair-wise blastn alignment gave 97% identities (752/779) and 2% gaps (15/779) [16]. Since, Ku et al. are giving experimental evidence for existence of a PCNA pseudogene on chromosome X [6], which is confirmed by Webb et al. [7] and the sequence of this PCNA pseudogene on chromosome X is virtually the same as the sequence of LOC392454, we suggest that LOC392454 is a valid PCNA pseudogene. As such, we recommend assignment of a name for LOC392454 as "proliferating cell nuclear antigen pseudogene 3" and the alias PCNAP3.

The LOC390102 is in the same locus as the PCNA pseudogene, described in Paper III and mapped on chromosome 11 (region 11p15.1). Webb et al. did not publish a sequence of the PCNA gene located on chromosome 11, however their method of detection proved to be valid for localisation of the PCNA gene and the PCNA pseudogene on chromosome X. Since the localisation of the PCNA pseudogene, described by Webb at al. is in the precise localisation region of LOC390102, and there are no known PCNA pseudogenes in close vicinity, we speculate that LOC390102 is indeed the PCNA pseudogene, detected by Webb et al. in 1990. If LOC390102 indeed represents a valid PCNA pseudogene, we recommend assignment of a name for LOC390102 as "proliferating cell nuclear antigen pseudogene 4" and the alias PCNAP4.

The priority of PCNAP3 to PCNAP4 is given on the basis of the first date of publication of the evidences for existence of the respective PCNA pseudogenes.

**Figure 2:**
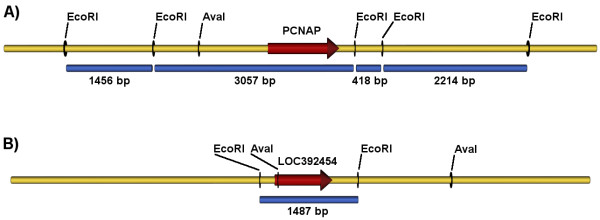
**Predicted EcoRI restriction pattern of the two loci on human chromosome X, containing a proposed PCNA pseudogene**. The restriction endonuclease sites of AvaI are denoted for additional clarity. A) PCNAP with additional 8 kb region (4 kb flanking in 5'- and 4 kb flanking in 3'-direction); B) LOC392454 with additional 8 kb region (4 kb flanking in 5'- and 4 kb flanking in 3'-direction). EcoRI (restriction endonuclease I from *Escherichia coli*), AvaI (restriction endonuclease I from *Anabaena variabilis*).

**Figure 3:**
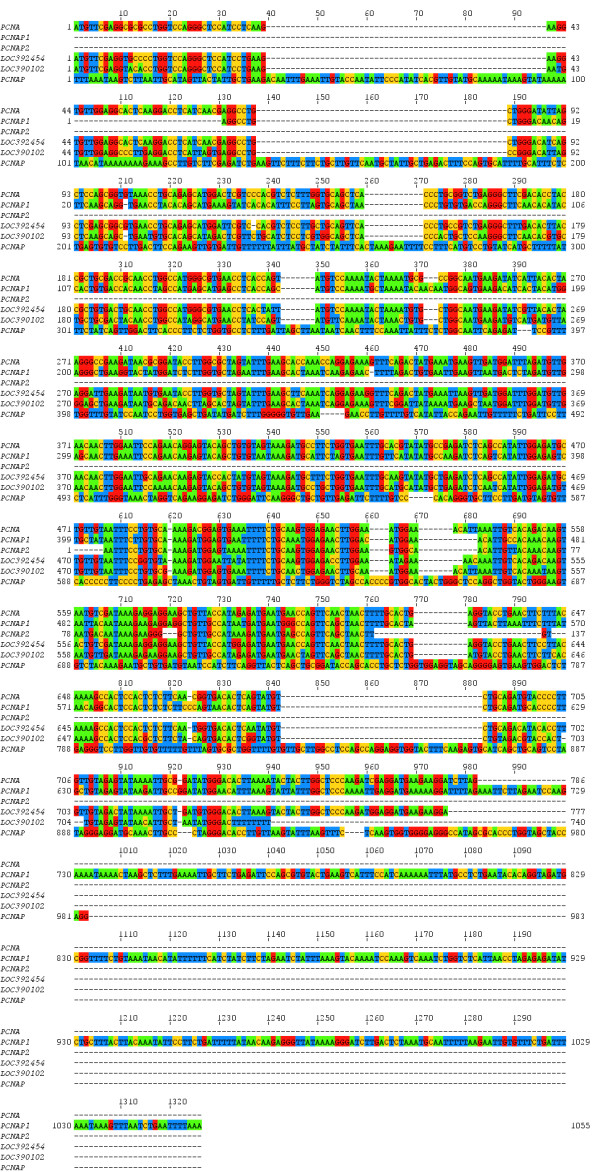
**Alignment of the sequences of the PCNA gene and the proposed PCNA pseudogenes (PCNAP1, PCNAP2, LOC392454, LOC390102 and PCNAP)**.

More problematic is the designation PCNAP, given by HUGO and mapped to the region Xp11. PCNAP is present in the NCBI GENE database as well. Both the HUGO database and the NCBI GENE database refer to the paper published by Ku et al. in 1989 [6]. There are several discrepancies between the sequence of PCNAP and the paper used for a reference. One is the observation made in Paper I of a restriction fragment of a size 1.5 kb, which hybridises with a probe based on a full length PCNA cDNA [5]. This fragment does not belong to the sequence of the PCNA gene and is proposed to represent a pseudogene [5]. Indeed, in the follow-up paper the same group detected this pseudogene, mapped it on chromosome X and presented its sequence [6]. As we mentioned earlier this sequence most probably represents LOC392454. We speculate that a restriction fragment of a size 1.5 kb, which is detected by a probe based on PCNA cDNA cannot be produced from the sequence of PCNAP given in NCBI GENE database (Figure 2). The aforementioned speculation is based on the sequences, available from human genome reference assembly GRC37 [17]. We used the sequence of PCNAP and its both flanking 4 kb regions in 3'- and 5'-direction, to predict the restriction pattern in a total EcoRI digest (Figure 2A). The sequence of PCNAP lies in a restriction fragment with a size of 3057 bp. A similar region of DNA, representing the LOC392454 and its 4 kb flanking regions in 3'- and 5'-direction, leaves LOC392454 in a hypothetical EcoRI restriction fragment with a size of 1487 kb (Figure 2B). The size of the predicted LOC392454 EcoRI restriction fragment is very similar to the size observed in Paper II and III. The other discrepancy we see is in the sequence of PCNAP. We aligned all proposed PCNA pseudogenes with the sequence of the PCNA gene, using mafft algorithm (Figure 3) [18]. We see little or no similarity between the sequences of the PCNAP and PCNA, as well as between the sequences of the PCNAP and the other PCNA pseudogenes. In contrast to PCNAP, the sequences of all other pseudogenes (PCNAP1, PCNAP2, LOC392454 and LOC390102) are aligning very well with the sequence of the PCNA gene. We performed a discontinuous megablast pair-wise alignment, using blastn algorithm, to score how each sequence of a proposed PCNA pseudogene aligns with the sequence of the PCNA gene [16]. The results are presented in Table 1. The sequence of PCNAP was the only one, which showed no significant similarity to the PCNA sequence. The other sequences were scored as having identities between 85% and 91% and gaps between 0% and 6% in respect to the PCNA gene sequence (Table 1). As we suggested above the PCNA pseudogene detected on human chromosome X by Ku et al. is analogous to LOC392454 and is not represented by the PCNAP sequence. We speculate that PCNAP might not represent a valid PCNA pseudogene.

**Table 1:** The result from a pair-wise alignment between the sequences of PCNA gene and the proposed PCNA pseudogenes

Pseudogene:	Identities towards PCNA	Gaps towards PCNA
**PCNAP1**	581/718 (81%)	12/718 (2%)

**PCNAP2**	122/143 (85%)	8/143 (6%)

**LOC392454**	711/780 (91%)	3/780 (0%)

**LOC390102**	629/736 (85%)	4/736 (1%)

**PCNAP**	N.S.	N.S.

Despite of the possibility of representing valid human PCNA pseudogenes, an interesting question is whether the sequences of PCNAP, PCNAP1, PCNAP2, LOC392454 and LOC390102 are present in the human trascriptome. In order to address this question, we checked for mRNA transcripts and expressed sequence tagged (EST) transcripts, associated with the corresponding sequences of the human PCNA pseudogenes. We were unable to find the sequences of any of the PCNA pseudogenes as stand-alone mRNA transcripts. However, when we checked the transcription profile of a respective genomic stretch in the UCSC genome browser [19], we found that a partial sequence of PCNAP1 is present in the A1223432, DA828011, FN062437 and CR743211 human EST transcripts. The sequence of LOC392454 was partially present in the human H79841 EST transcript. There are no ESTs matches with the sequences of PCNAP, PCNAP2 or LOC390102. Despite of the existence of EST transcripts which matched the sequence of the PCNA pseudogenes, we would like to express caution in the interpretation of such results. ESTs libraries are prone to non-mRNA contamination including genomic DNA, and it is possible that the regions of interest are not transcribed at all [20]. We also performed a BLAST search in the human ESTs database targeting the sequences of the proposed PCNA pseudogenes, and although we detected matches we could not conclude with certainty if the sequences of the PCNA pseudogenes are transcribed or not.

## Conclusions

In summary we propose the existence of at least four valid PCNA pseudogenes, PCNAP1, PCNAP2, LOC392454 and LOC390102. We would like to recommend assignment of a name for LOC392454 as "proliferating cell nuclear antigen pseudogene 3" (alias PCNAP3) and a name for LOC390102 as "proliferating cell nuclear antigen pseudogene 4" (alias PCNAP4). We prompt for more critical evaluation of the existence of a PCNA pseudogene, designated as PCNAP.

## Abbreviations

EcoRI: restriction endonuclease I from *Escherichia coli*; HUGO: Human Genome Organisation; LOC390102: locus 390102; LOC392454: locus 392454; NCBI: National Center for Biotechnology Information; PCNA: Proliferating Cell Nuclear Antigen; PCNAP: Proliferating Cell Nuclear Antigen Pseudogene; PCNAP1: Proliferating Cell Nuclear Antigen Pseudogene 1; PCNAP2: Proliferating Cell Nuclear Antigen Pseudogene 2.

## Competing interests

The authors declare that they have no competing interests.

## Authors' contributions

IS designed the study, acquired and analysed the human genome data. AL critically evaluated the results. IS and AL participated equally in the writing of the manuscript. Both authors read and approved the final manuscript.
